# Aspartic Acid Residue 51 of SaeR Is Essential for *Staphylococcus aureus* Virulence

**DOI:** 10.3389/fmicb.2018.03085

**Published:** 2018-12-14

**Authors:** Tyler K. Nygaard, Timothy R. Borgogna, Eli W. Sward, Fermin E. Guerra, Jennifer G. Dankoff, Madison M. Collins, Kyler B. Pallister, Liang Chen, Barry N. Kreiswirth, Jovanka M. Voyich

**Affiliations:** ^1^Department of Microbiology and Immunology, Montana State University, Bozeman, MT, United States; ^2^Public Health Research Institute Tuberculosis Center, New Jersey Medical School, Rutgers University, Newark, NJ, United States

**Keywords:** *Staphylococcus aureus*, *saeR/S*, virulence, pathogenesis, two-components system, neutrophil, toxin, transcription

## Abstract

*Staphylococcus aureus* is a common Gram-positive bacteria that is a major cause of human morbidity and mortality. The SaeR/S two-component sensory system of *S. aureus* is important for virulence gene transcription and pathogenesis. However, the influence of SaeR phosphorylation on virulence gene transcription is not clear. To determine the importance of potential SaeR phosphorylation sites for *S. aureus* virulence, we generated genomic alanine substitutions at conserved aspartic acid residues in the receiver domain of the SaeR response regulator in clinically significant *S. aureus* pulsed-field gel electrophoresis (PFGE) type USA300. Transcriptional analysis demonstrated a dramatic reduction in the transcript abundance of various toxins, adhesins, and immunomodulatory proteins for SaeR with an aspartic acid to alanine substitution at residue 51. These findings corresponded to a significant decrease in cytotoxicity against human erythrocytes and polymorphonuclear leukocytes, the ability to block human myeloperoxidase activity, and pathogenesis during murine soft-tissue infection. Analysis of SaeR sequences from over 8,000 draft *S. aureus* genomes revealed that aspartic acid residue 51 is 100% conserved. Collectively, these results demonstrate that aspartic acid residue 51 of SaeR is essential for *S. aureus* virulence and underscore a conserved target for novel antimicrobial strategies that treat infection caused by this pathogen.

## Introduction

*Staphylococcus aureus* (*S. aureus*) is a Gram-positive bacterium that can cause a wide range of disease in both humans and animals (Nygaard et al., 2008). The diverse pathogenesis of *S. aureus* can be attributed to the expression of an extensive array virulence factors that are often redundant in function (Guerra et al., 2017). Expression of these virulence genes *in vivo* is thought to be primarily dictated by the concerted influence of two-component sensory systems that recognize environmental signals and alter gene expression accordingly. The *S. aureus* genome encodes 16 two-component systems that have been identified by sequence analysis (Cheung et al., 2004). Of these, the SaeR/S two-component system has been shown to be an important mediator of *S. aureus* virulence by transcriptionally upregulating numerous adhesins, toxins, and immunomodulatory proteins that interact directly with host components to advance pathogenesis (Giraudo et al., 1997; Goerke et al., 2001; Voyich et al., 2009; Nygaard et al., 2010; Borgogna et al., 2018). The mechanisms used by this two-component system to recognize host-specific cues and alter gene transcription in response are not completely understood.

Typical two-component systems are minimally composed of a transmembrane histidine kinase sensor and cognate intracellular response regulator (Groisman, 2016). Recognition of environmental stimulus activates the histidine kinase sensor, inducing autophosphorylation at a histidine residue on the intracellular domain. This phosphate group is then transferred to an aspartic acid residue within the receiver domain of the cognate response regulator. In general, the phosphorylated response regulator then binds to promoter regions within the bacterial genome to mediate gene transcription and promote survival. However, some response regulators such as RcsB from *Escherichia coli* (Pannen et al., 2016) and SreR from *Xanthomonas campestris* (Wang et al., 2014) can regulate gene transcription without being phosphorylated. For the SaeR/S two-component system, the need to artificially phosphorylate recombinant SaeR for the *in vitro* DNA binding activity of this response regulator has not been consistent between studies (Nygaard et al., 2010; Sun et al., 2010). It has also been suggested that different levels of SaeR phosphorylation correspond to the upregulation of distinct groups of SaeR/S regulated genes (Mainiero et al., 2010).

To clarify the importance of potential SaeR phosphorylation sites for mediating *S. aureus* pathogenesis, we have generated individual single amino acid substitutions at conserved aspartic acid residues of SaeR in the genome of *S. aureus* PFGE-type USA300. In this report, we assess the significance of these residues for mediating the virulence of a clinically relevant MRSA strain using both *in vitro* and *in vivo* models of infection. Results from this study underscore the importance of SaeR aspartic acid residue 51 for virulence gene transcription and demonstrate that the substitution of this single amino acid in the USA300 genome can attenuate pathogenesis of this clinically significant MRSA strain.

## Materials and Methods

### Bacteria Strains and Culture Conditions

*Staphylococcus aureus* PFGE-type USA300 strain LAC has been described previously (Diep et al., 2006) and the USA300 isogenic deletion mutant of *saeR/S* (USA300Δ*saeR/S*) was generated in previous studies (Nygaard et al., 2010). Bacteria were cultured in an Excella E24 rotary incubator (New Brunswick) at 250 rpm and 37°C. Unless noted otherwise, overnight bacteria cultures grown in 20 mL of tryptic soy broth (TSB; EMD Millipore) were used to start subcultures in 20 mL TSB containing 0.5% glucose (1:100 dilution). Optical density at 600 nm (OD_600_) was measured using a NanoDrop 2000c Spectrophotometer (ThermoFisher Scientific) and colony forming units (CFUs) were determined by plating diluted samples on tryptic soy agar (TSA; EMD Millipore).

### DNA Sequence Alignment

DNA sequence alignment of SaeR homologs was performed using Clustal Omega^1^ with protein sequences obtained from the NCBI protein data base using the following accession numbers: ABD22784 for USA300, ATX72322 for USA400, EOR90509 for USA100, WP_076742615.1 for MT-0541, AAW53763 for *Staphylococcus epidermidis* RP62A, Q8CQ17 for *Staphylococcus epidermidis* 12228, WP_049307325 for *Staphylococcus capitis*, WP_029378577 for *Staphylococcus xylosus*, PCQ20359 for *Klebsiella pneumonia*, NP_346930 for *Clostridium acetobutylicum*, ACJ50526 for *Escherichia coli*, and NP_388082 for *Bacillus subtilis*.

### Generation of USA300 Genomic Mutations

Allelic exchange with plasmid pKOR1 was used to impart genomic mutations in USA300 as previously described (Bae and Schneewind, 2006). All primers used to generate and sequence USA300 mutants are listed in Supplementary Table S1. Primers containing *attb* sites were used to amplify staphylococcal peroxidase inhibitor (*spn*) and saeR from the USA300 genome for BP Clonase II (ThermoFisher Scientific) mediated insertion into pKOR1. Spn-check-fwd and rvs primers were used to verify the loss of *spn*. Site-directed mutagenesis was performed on pKOR1-*saeR* using saeR-D46A, saeR-D51A, or saeR-D61A primers and PfuUltra II Fusion HS DNA polymerase (Agilent Technologies, Inc.) following the manufacture’s protocol and as previously performed (Ran et al., 2010). The *saePQRS* operon in generated *saeR* point mutants was PCR amplified using saePQRS_fwd and rvs primers and sequenced (BigDye Terminator v3.1 Cycle Sequencing Kit) using saeR-seq and saePQRS-seq primers. PCR amplification was performed using saeR-EcoRI-fwd and saeR-XhoI-rvs and cloned into pEPSA5 as previously described (Flack et al., 2014) to generate the complementary plasmid that expresses wt SaeR, pEPSA5-*saeR*.

### Whole Genome Sequencing and *saeR* Sequence Analysis

Wild-type USA300 and its SaeR mutants were subject to whole genome sequencing. Briefly, genomic DNA was extracted using a Wizard genomic DNA purification kit (Promega, Madison, WI, United States) following by treatment with 20 μg/ml lysostaphin. The DNA library was sequenced on an Illumina NextSeq platform (Illumina, San Diego, CA, United States) with 2 × 150 bp paired-end reads. The reads were mapped against the published NC_007793 (*S. aureus* USA300_FPR3757) genome using BWA (Li and Durbin, 2009) and Samtools (Li et al., 2009), and the SNPs and InDels were examined using freebayes^2^. SNPs and InDels were further annotated using snpEff (Cingolani et al., 2012).

Blast analysis of the USA300 *saeR* (locus_tag SAUSA300_0691) sequence against more than 8,000 *S. aureus* draft genomes downloaded from the NCBI FTP site^3^ (dated as June 1, 2018) were extracted, translated into amino acids and aligned using Geneious 11.1.

### Relative Quantitative Real Time RT-PCR

*Staphylococcus aureus* transcription analysis using relative quantitative real time RT-PCR was performed as previously described (Voyich et al., 2005, 2009; Nygaard et al., 2010). Briefly, subcultured strains were harvested at mid-exponential (ME; OD_600_ = 1.5) or early stationary (ES; OD_600_ = 3.0) growth phases, mechanically disrupted using a FastPrep FP120 cell disrupter (ThermoFisher Scientific), and RNA purified with an RNeasy Kit (Qiagen). TaqMan real-time RT-PCR was performed using previously published primer and probe sets (Nygaard et al., 2010).

### Hemolysis Assays

Heparinized venous blood from healthy donors was collected in accordance with the protocol approved by the Institutional Review Board for Human Subjects at Montana State University. All donors provided written informed consent to participate in the study. We adopted protocol described by others (Young et al., 1986) to quantify hemolysis of human blood by *S. aureus* extracellular proteins. Briefly, freshly drawn human blood was washed three times with 10 times the volume of sterile DPBS then resuspended at a final dilution of 1:200 with sterile DPBS. Sterile-filtered (0.22 um) *S. aureus* supernatants from 6-h subcultures grown in TSB were combined with washed diluted blood in individual wells of a 96 well plate on ice at a ratio of 1:1. TSB alone and TSB + 0.5% Triton X-100 were also used as negative and positive controls for hemolysis. Samples were then placed in a SpectraMax 190 microplate reader (Molecular Devices) heated to 37°C and absorbance at 630 nm was measured after 6 min of incubation. Percent hemolysis was determined using the following formula: %Hemolysis = (Absorbance_Experimental_ – Absorbance _TSBcontrol_)/(Absorbance_Triton X_ – Absorbance _TSBcontrol_) × 100.

### Human Myeloperoxidase Activity Assays

To obtain extracellular proteins from USA300 strains, supernatant from *S. aureus* sub-cultured for 5 h was sterile-filtered (0.22 um) and stored at -80°C. For human myeloperoxidase (MPO) activity assays, 0.5 ug of recombinant human MPO (R&D Systems) in 50 uL of DPBS was mixed with 50 uL of sterile-filtered *S. aureus* supernatant for 30 min at room temperature. The MPO-supernatant solution was then exposed to 150 uL of 3,3′,5,5′-tetramethylbenzidine (TMB) substrate reagent set (BD Biosciences) and incubated at 37°C. The oxidation of TMB catalyzed by human MPO was quantified by measuring the OD_650_ every minute for 30 min using a SpectraMax Paradigm microplate reader (Molecular Devices). The MPO inhibitor sodium azide was used at a concentration of 1 mM.

### Human PMN Plasma Membrane Integrity Assays

Human polymorphonuclear leukocytes (neutrophils or PMNs) were isolated under endotoxin-free conditions (<25.0 pg/ml) using freshly drawn heparinized venous blood from healthy donors with written informed consent as previously described (Voyich et al., 2005; Nygaard et al., 2013). Cell viability and purity of preparations were assessed using a FACSCalibur Flow cytometer (BD Biosciences) and only preparations containing ≥ 98% viable PMNs were used. Assays intoxicating PMNs with extracellular *S. aureus* proteins were performed as previously described (Nygaard et al., 2012; Flack et al., 2014). Briefly, supernatant from *S. aureus* subcultured for 5 h in TSB was sterile-filtered (0.22 um) and diluted by 1:10 with TSB. To intoxicate PMNs, 20 ul of diluted *S. aureus* supernatant was combined with 100 uL Roswell Park Memorial Institute (RPMI) 1640 Medium (Corning Cellgro) with 5 mM 4-(2-hydroxyethyl)-1-piperazineethanesulfonic acid (HEPES; Corning Cellgro) containing 1x10^6^ purified human PMNs in a serum coated well of a 96 well plate. Samples were incubated at 37°C for 90 min then stained with propidium iodide (PI; ThermoFisher Scientific) following the manufactures protocol and then analyzed with a FACSCalibur Flow cytometer. Triton-X 100 (0.5%) was used as a positive control for causing PMN plasma membrane permeability. The following formula was used to determine % propidium iodide^+^: %Propidium Iodide^+^ = (Mean PI signal_Experimental_ – Mean PI signal _untreated_)/(Mean PI signal_Triton-X_ – Mean PI signal _untreated_).

Assays measuring human PMN plasma membrane permeability following the phagocytosis of live *S. aureus* were performed as previously described (Voyich et al., 2009; Nygaard et al., 2012; Flack et al., 2014). Briefly, subcultured *S. aureus* was harvested at ME growth by centrifugation (5,000 × g, 5 min, 4°C), washed with DPBS, then opsonized with 50% normal human serum for 15 min at 37°C. Opsonized bacteria were washed with DPBS and then 2 × 10^7^ CFU in 100 uL of DPBS was combined with 100 uL of RPMI/H containing 1 × 10^6^ purified human PMNs in a serum coated well of 96 well plate. Phagocytosis was synchronized by centrifugation (524 × g, 8°C, 8 min) in an Allegra X-15R centrifuge (Beckman Coulter) and samples were incubated at 37°C for 90 min. Following incubation, human PMNs were stained with PI and analyzed using flow cytometry as described above.

### Murine Model of Soft-Tissue Infection

All animal studies conformed to National Institute of Health guidelines and were approved by the Animal Care and Use Committee at Montana State University-Bozeman. Female BALB/C mice (8–10 weeks old) with an average weight of 22 g were purchased from Animal Resource Facility at Montana State University (Bozeman, MT, United States). The murine model of soft-tissue infection was performed as previously described (Voyich et al., 2006; Nygaard et al., 2010; Malachowa et al., 2013). Briefly, 12-week-old BALB/C mice were shaved and hair completely removed with Nair^TM^ treatment. Two days later, shaved mice (5 per group) were subcutaneously inoculated with 1 × 10^7^ CFUs of *S. aureus* in 100 uL DPBS. Mice were weighed and abscess size measured at indicated times post-inoculation. The area of soft-tissue infections was determined using the following formula as previously published (Malachowa et al., 2013): Area = π (Length/2) × Width/2.

## Results

### The Generation of Point Mutations in the USA300 Genome That Confer Aspartic Acid to Alanine Substitutions Within the Receiver Domain of SaeR

**FIGURE 1:**
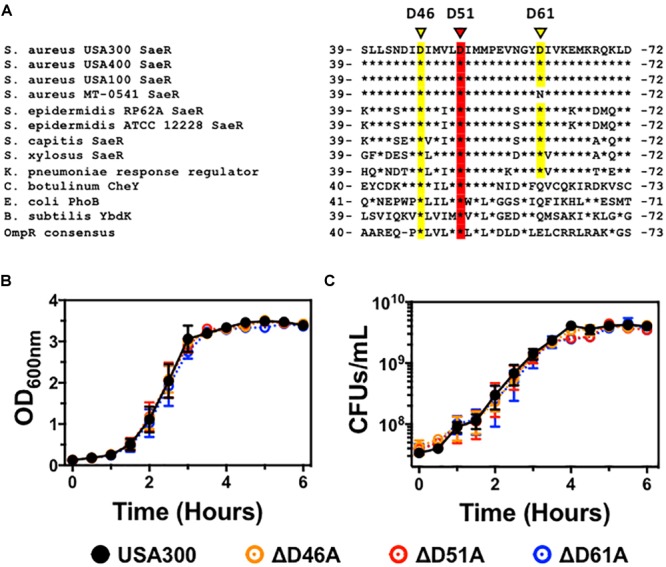
The generation of USA300 genomic point mutations in specific aspartic acid residues of *saeR.* **(A)** DNA sequence alignment of *saeR* homologues from different bacteria highlighting the putative phosphorylation site at conserved aspartic acid residue 51 (red) as well as proximal aspartic acid residues 46 and 61 (yellow). *In vitro* growth as measured by **(B)** absorbance at 600 nm, and **(C)** colony forming units (CFU) per mL of USA300, USA300 with an aspartic acid to alanine point mutation at SaeR residue 46 (ΔD46A), at residue 51 (ΔD51A), or at residue 61 (ΔD61A). Data in panels **(B,C)** are presented as the mean ± SEM of three independent experiments.

Analysis of the three-dimensional crystal structure of the two-component response regulator PhoB of *Escherichia coli* (Solà et al., 1999) indicates that the homologous *S. aureus* response regulator SaeR is phosphorylated on a conserved aspartic acid at residue 51 (Figure 1A). Aspartic acid residue 46 is also highly conserved in the OmpR family of proteins, suggesting this residue might be important for SaeR function. Analysis of the SaeR sequences extracted from over 8,000 draft *S. aureus* genomes revealed that aspartic acid residue 51 is 100% conserved. The biological significance of this conservation is heightened by the identification of 50 unique SaeR protein sequences identified from the draft genomes (Supplementary Figure S1).

To determine if these aspartic acid residues in the receiver domain of SaeR are important for *S. aureus* pathogenesis, we used allelic recombination to induce substitutions of specific nucleic acids within the *S. aureus* genome as previously described by others (Bae and Schneewind, 2006; Villaruz et al., 2009; Mairpady Shambat et al., 2016). Using this technique, we generated USA300 strains with an aspartic acid to alanine substitution at the conserved SaeR residue 51 (USA300Δ*saeR*-D51A) or the proximal conserved residue 46 (USA300Δ*saeR*-D46A). In addition, we generated an aspartic acid to alanine substitution at the less maintained residue 61 of SaeR (USA300Δ*saeR*-D61A) to serve as a control for this study. Primary DNA sequencing analysis and additional whole genome sequencing analysis verified the targeted amino acid substitutions that were generated in the three mutants and showed that there were no additional SNPs or InDels when compared to the wild-type strain. As with previously generated isogenic deletion mutants of *saeR/S* in *S. aureus* (Voyich et al., 2009; Nygaard et al., 2010), no differences during *in vitro* growth could be detected in these SaeR point mutants relative to the parental USA300 strain (Figures 1B,C).

### Substitution of SaeR Aspartic Acid Residue 51 With Alanine Substantially Decreases the Transcript Abundance of USA300 Virulence Genes

The SaeR/S two-component system plays an essential role during pathogenesis by upregulating *S. aureus* virulence gene transcription (Giraudo et al., 1997; Goerke et al., 2001; Voyich et al., 2009; Nygaard et al., 2010). It is thought that this process requires the activation of SaeR via phosphorylation at an aspartic acid residue within the receiver domain of this response regulator. Indeed, others have indicated that aspartic acid residue 51 of SaeR is necessary for the transcription of α-hemolysin (Hla) using an artificial plasmid overexpression system in *S. aureus* strain Newman lacking wild-type *saeR/S* (Mainiero et al., 2010). However, the transcription of *saeR* that is under a positive feedback loop by the SaeR/S two-component system was actually enhanced in the absence of aspartic acid 51 while no difference in SaeR/S regulated coagulase A transcription could be demonstrated (Mainiero et al., 2010). This study suggested that different levels of SaeR phosphorylation corresponds to the up-regulation of specific sets of virulence genes, prompting us to also examine the influence of the highly conserved SaeR aspartic acid residue 46 and partially conserved aspartic acid residue 61 on SaeR/S-mediated virulence gene transcription.

**FIGURE 2:**
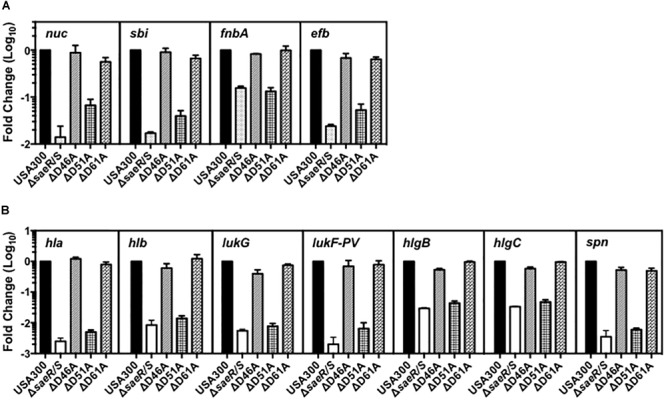
Aspartic acid residue 51 of SaeR is important for the transcription of numerous virulence genes in USA300. Taqman^®^ RT-PCR analysis of USA300, an isogenic deletion mutant of *saeR/S* in USA300 (USA300Δ*saeR/S*), and USA300 genomic point mutants that confer aspartic acid to alanine substitutions at SaeR residue 46 (ΔD46A), 51 (ΔD51A), or 61 (ΔD61A) during growth *in vitro*. Transcriptional analysis was performed at **(A)** mid-exponential growth for nuclease (*nuc)*, the second binder of IgG (*sbi*), fibronectin-binding protein A (*fnbA)*, and the extracellular fibrinogen-binding protein (*efb*) or at **(B)** early-stationary growth for γ-hemolysin (*hla*), γ-hemolysin (*hlb*), leukocidin subunit G (*lukG)*, the Panton-Valentine leukocidin subunit F (*lukF-PV*), γ-hemolysin component B (*hlgB)*,γ-hemolysin component C (*hlgC)*, and the staphylococcal peroxidase inhibitor (*spn*). All panels show the mean ± SEM of at least two separate experiments and are presented as fold change relative to USA300 wt.

To resolve the importance of these SaeR aspartic acid residues for virulence gene expression in clinically relevant MRSA, we examined the transcript abundance of numerous adhesins, toxins, and immunomodulatory proteins in the USA300 aspartic acid point mutant strains using relative quantitative real time RT-PCR (Figure 2). Compared to USA300, we observed substantial decreases in the abundance of transcripts encoding various adhesins, toxins, and immunomodulatory proteins in a USA300 isogenic deletion mutant of *saeR/*S (USA300Δ*saeR/S*) relative to the USA300 wt (Figures 2A,B). The virulence gene transcription profile for USA300Δ*saeR*-D51A was almost identical to that of USA300Δ*saeR/S*. In contrast, the transcript abundance of virulence genes in USA300Δ*saeR*-D46A and USA300Δ*saeR*-D61A was comparable to the USA300Δ parental wt (Figures 2A,B). Reintroduction of wild-type SaeR to USA300Δ*saeR*-D51A rescued defects in gene transcription (Supplementary Figure S2), supporting results indicating aspartic acid residue 51 of SaeR is required for the upregulation of virulence gene expression by this response regulator.

### Aspartic Acid Residue 51 of SaeR Is Essential for the SaeR/S Mediated Hemolysis of Human Erythrocytes by USA300

**FIGURE 3:**
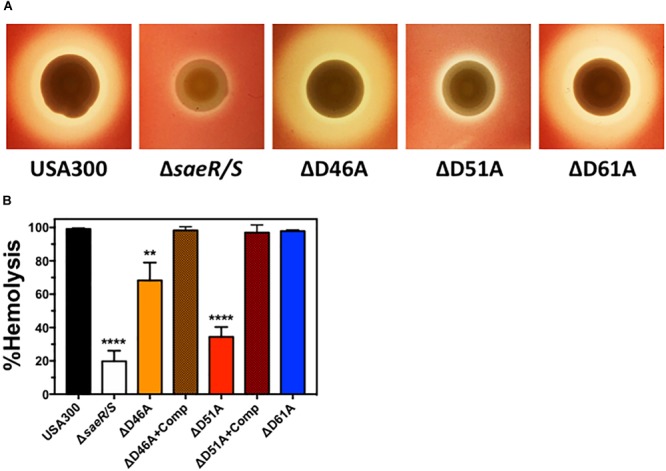
Aspartic acid residues 46 and 51 of SaeR are important for the hemolysis of human erythrocytes by USA300. **(A)** Zone of hemolysis caused by USA300, an isogenic deletion mutant of *saeR/S* in USA300 (USA300Δ*saeR/S*), or USA300 expressing SaeR with aspartic acid to alanine substitutions at residue 46 (ΔD46A), 51 (ΔD51A), or 61 (ΔD61A) during growth on agar containing 2.5% human red blood cells. **(B)** Percent hemolysis of human red blood cells in solution exposed to 6 h supernatants from USA300, USA300Δ*saeR/S*, ΔD46A, ΔD51A, ΔD61A or ΔD46A and ΔD51A complemented with a plasmid expressing wt SaeR (ΔD46A+Comp or ΔD51A+comp, respectively). All panels represent at least 3 separate experiments. Panels **(B,C)** show the mean ± SEM with ^∗∗^*P* ≤ 0.01 and ^∗∗∗∗^*P* ≤ 0.0001 relative to USA300 as determined by one-way ANOVA with Dunnett’s multiple comparison test.

*Staphylococcus aureus* expresses numerous hemolysins under direct transcriptional regulation by the SaeR/S two-component system (Voyich et al., 2009; Nygaard et al., 2010) that lyse mammalian erythrocytes including Hla, Hlb, and HlgA/B (Salgado-Pabón et al., 2014; Spaan et al., 2015). Real time RT-PCR analysis indicated aspartic acid residue 51 of SaeR is essential for the transcriptional upregulation of these hemolysins (Figure 2). To determine the importance of this putative SaeR phosphorylation site for hemolysis caused by *S. aureus*, we first qualitatively assessed the ability of USA300, USA300Δ*saeR/S*, USA300Δ*saeR*-D46A, USA300Δ*saeR*-D51A, and USA300Δ*saeR*-D61A to lyse human red blood cells during growth on agar (Figure 3A). The zone of hemolysis generated by USA300Δ*saeR/S* on agar was decreased relative to USA300 (Figure 3A), corresponding to observations in this study (Figure 2) and in previously published reports (Voyich et al., 2009; Nygaard et al., 2010) that demonstrate a decrease in transcript abundance of various *S. aureus* hemolysins when this two-component system is absent. A reduced hemolysis of human erythrocytes during growth on agar of USA300Δ*saeR*-D51A paralleled that of USA300Δ*saeR/S*, supporting the notion that aspartic acid residue 51 is essential for SaeR/S activity. In contrast, the zone of hemolysis for USA300Δ*saeR*-D46A and USA300Δ*saeR*-D61A was indistinguishable from that caused by USA300.

To quantify the hemolysis caused by aspartic acid point mutants of SaeR, we measured the turbidity of human erythrocytes in solution after being combined with extracellular proteins produced by these strains. Corresponding to hemolysis during growth on agar, significantly less human red blood cells were lysed by filtered supernatants from USA300Δ*saeR*/S or USA300Δ*saeR*-D51A relative to the USA300 parental strain (Figure 3B). In addition, extracellular proteins produced by USA300Δ*saeR*-D46A generated significantly less hemolysis than USA300. In support of these findings, the reintroduction of wild-type SaeR to USA300Δ*saeR*-D46A or USA300Δ*saeR*-D51A rescued the hemolytic activity of supernatants from these strains to levels observed for USA300. Taken together, these findings show that aspartic acid residue 51 of SaeR is necessary for causing the hemolysis of human erythrocytes that is facilitated by the SaeR/S two-component system. In addition, SaeR aspartic acid residue 46 appears to play a significant but less important role than residue 51 mediating hemolysis caused by USA300.

### Inhibition of Human MPO Activity by USA300 Extracellular Proteins Requires Aspartic Acid Residue 51 of SaeR

**FIGURE 4:**
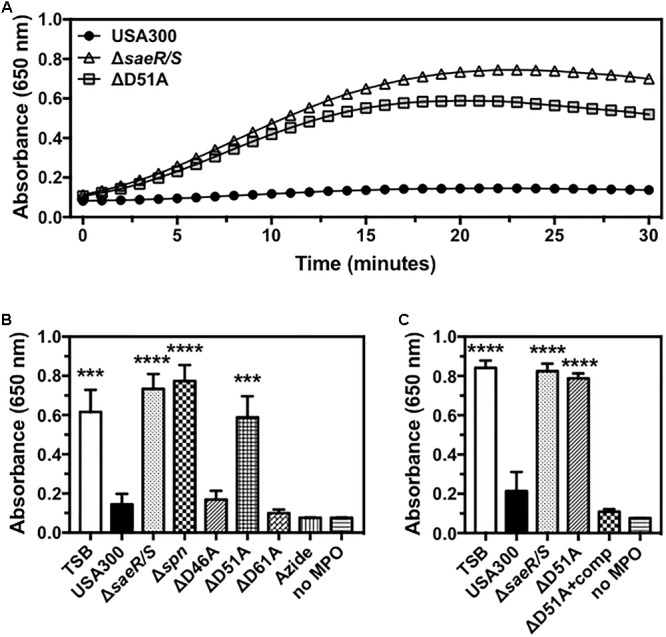
Aspartic acid residue 51 of SaeR is essential for the inhibition of human myeloperoxidase activity by USA300. **(A)** Human myeloperoxidase (MPO) activity in the presence of extracellular proteins produced by USA300, an isogenic deletion mutant of *saeR/S* in USA300 (Δ*saeR/S*), or an aspartic acid to alanine substitution at residue 51 of SaeR in USA300 (ΔD51A) as measured by absorbance at 650 nm following exposure to 3,3′,5,5′-Tetramethylbenzidine (TMB) and hydrogen peroxide. **(B)** The activity of human MPO in the presence of extracellular proteins produced by USA300, Δ*saeR/S*, an isogenic deletion mutant of SPIN in USA300 (Δ*spn*), ΔD51A, or USA300 with an aspartic acid to alanine substitution at residue 46 (ΔD46A) or 61 (ΔD61A) of SaeR. Peroxidase activity in the presence of the MPO inhibitor sodium azide (Azide) or samples lacking MPO (no MPO) were included as negative controls. **(C)** Experiments in panel **(B)** were repeated using a D51A point mutant of *saeR* complemented with a plasmid expressing wt SaeR (ΔD51A+comp). All panels show the mean ± SEM of at least three independent experiments. For panels **(B,C)**, absorbance is shown at 20 min with ^∗∗∗^*P* < 0.001 and ^∗∗∗∗^*P* < 0.0001 relative to USA300 as determined by one-way ANOVA with Dunnett’s multiple comparisons test.

Recently published findings demonstrate that *S. aureus* prevents the generation of reactive oxygen species by human polymorphonuclear leukocytes (neutrophils or PMNs) via SPIN, a protein under strong SaeR/S regulation that binds directly to the active site of human MPO and inhibits peroxidase activity of this enzyme (Guerra et al., 2016; de Jong et al., 2017). To determine if the putative phosphorylation site at aspartic acid residue 51 of SaeR is required for the inhibition of MPO activity by USA300, we assessed the activity of human MPO in the presence of extracellular proteins produced by USA300, an isogenic deletion mutant of SPIN in USA300 (USA300Δ*spn*), USA300Δ*saeR/S*, USA300Δ*saeR*-D46A, USA300Δ*saeR*-D51A, and USA300Δ*saeR*-D61A (Figure 4). Congruent with previously published findings (de Jong et al., 2017), a strong inhibition of human MPO activity was observed in the presence of the MPO inhibitor azide or extracellular proteins produced by USA300 but not extracellular proteins produced by USA300Δ*saeR/S* or by USA300Δ*spn* (Figures 4A,B). As with USA300Δ*saeR/S* and USA300Δ*spn*, extracellular proteins produced by USA300Δ*saeR*-D51A did not inhibit human MPO activity (Figures 4A,B). Reintroduction of wild-type SaeR to USA300Δ*saeR*-D51A rescued the inhibition of human MPO activity by extracellular proteins produced from this strain (Figure 4C). In contrast, supernatant from USA300Δ*saeR*-D46A and USA300Δ*saeR*-D61A blocked the activity of human MPO.

### Human PMN Plasma Membrane Permeability Caused by USA300 Requires Aspartic Acid at Residue 51 of SaeR

**FIGURE 5:**
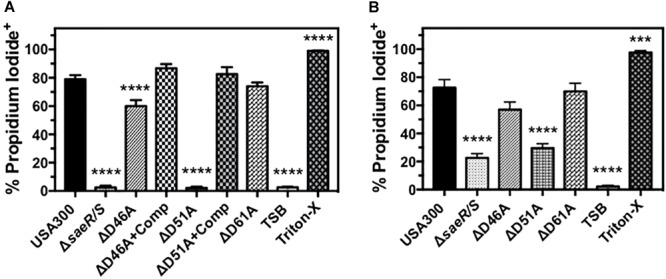
Human PMN plasma membrane permeability caused by USA300 is significantly influenced by aspartic acid residues 46 and 51 of SaeR. **(A)** Flow cytometry was used to assess the percentage of purified human PMNs permeable to propidium iodide 90 min after exposure to 5 h supernatants from USA300, an isogenic deletion mutant of *saeR/S* in USA300 (USA300Δ*saeR/S*), or USA300 expressing SaeR with aspartic acid to alanine substitutions at residue 46 (ΔD46A), 51 (ΔD51A), 61 (ΔD61A), and ΔD46A or ΔD51A complemented with plasmids expressing wt SaeR (ΔD46A+Comp or ΔD51A+comp, respectively). **(B)** The percentage of propidium iodide positive human PMNs 90 min after the phagocytosis of live *Staphylococcus aureus* at a ratio of 20 colony forming units per PMN. For these experiments, PMNs were also treated with tryptic soy broth (TSB) alone or TSB with 0.5% Triton X-100 (Triton-X). All panels show the mean ± SEM of at least 5 independent experiments. ^∗∗∗^*P* ≤ 0.001 and ^∗∗∗∗^*P* ≤ 0.0001 relative to USA300 as determined by one-way ANOVA with Dunnett’s multiple comparison test.

Previous studies have demonstrated that the SaeR/S two-component system upregulates *S. aureus* toxins that target human PMNs (Gauduchon et al., 2001; Voyich et al., 2009; Nygaard et al., 2010; Ventura et al., 2010; DuMont et al., 2011; Malachowa et al., 2011). To assess the importance of aspartic acid residues within the receiver domain of SaeR for mediating toxicity against PMNs, we first examined the ability of extracellular proteins produced by D46A, D51A, and D61A point mutants of SaeR to cause human PMN plasma membrane permeability as measured by propidium iodide staining (Figure 5A). No plasma membrane permeability was observed for PMNs exposed to extracellular proteins produced by USA300Δ*saeR*-D51A, indicating aspartic acid residue 51 of SaeR is necessary for the toxicity of USA300 against human PMNs. A significant reduction in PMN plasma membrane permeability was also observed for extracellular proteins produced by USA300Δ*saeR*-D46A, though this difference was less than that observed for USA300Δ*saeR*-D51A. In support of these findings, complementation of USA300Δ*saeR*-D46A, or USA300Δ*saeR*-D51A with wild-type SaeR rescued the lytic capacity of extracellular proteins produced by these strains against human PMNs. Corresponding to the toxicity of extracellular proteins produced by these strains, significantly less PMN plasma membrane permeability was observed following phagocytosis of live USA300Δ*saeR/S*, USA300Δ*saeR*-D51A, and USA300Δ*saeR*-D46A relative to USA300 (Figure 5B). These results demonstrate that SaeR aspartic acid residue 51 and, to a lesser extent, aspartic acid residue 46 are important for activity of this two-component system.

### Aspartic Acid Residue 51 of SaeR Is Essential for the Pathogenesis of USA300 During Murine Soft-Tissue Infections

**FIGURE 6:**
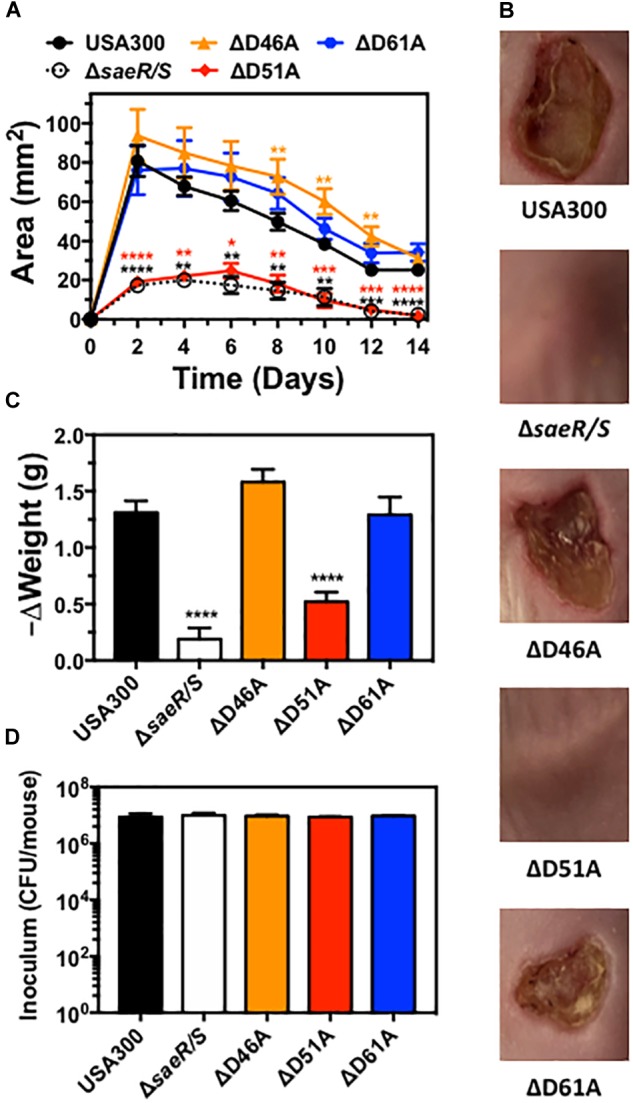
Aspartic acid residue 51 of SaeR significantly influences the pathogenesis of murine soft-tissue infections caused by USA300. **(A)** Area of soft-tissue infection caused by USA300, an isogenic deletion mutant of *saeR/S* in USA300 (Δ*saeR/S*), or USA300 with an aspartic acid to alanine substitution at SaeR residue 46 (ΔD46A), 51 (ΔD51A) or 61 (ΔD61A). **(B)** Representative images of soft-tissue infections from experiments in panel **(A)** on day 6 post-inoculation. **(C)** Change in the weight of mice during experiments in panel **(A)** between days 0 and 2 post-inoculation. **(D)** Inoculum of each strain given to mice in panels **(A-C)**. Panels **(A), (C)**, and **(D)** show the mean ± SEM of two independent experiments. ^∗^*P* ≤ 0.05, ^∗∗^*P* ≤ 0.01, ^∗∗∗^*P* ≤ 0.001 and ^∗∗∗∗^*P* ≤ 0.0001 relative to USA300 as determined by one-way ANOVA with Dunnett’s multiple comparison test.

Previously published findings demonstrate the upregulation of virulence gene transcription *in vivo* by the SaeR/S two-component system is essential for the pathogenesis of *S. aureus* during both systemic and localized infection (Voyich et al., 2009; Nygaard et al., 2010). Transcriptional analysis of USA300Δ*saeR*-D51A in this report (Figure 2) indicates aspartic acid residue 51 of SaeR is required for upregulating *S. aureus* virulence gene transcription mediated by SaeR/S *in vitro*. To determine if potential SaeR phosphorylation sites are important for pathogenesis *in vivo*, we assessed the virulence of USA300, USA300Δ*saeR/S*, USA300Δ*saeR*-D46A, USA300Δ*saeR*-D51A, and USA300Δ*saeR*-D61A during a murine model of soft-tissue infection (Figure 6). In a manner indistinguishable from USA300Δ*saeR/S*, soft-tissue infections caused by USA300Δ*saeR*-D51A were significantly smaller than infections caused by USA300 (Figure 6A) and did not exhibit the open dermonecrotic lesions characteristic of USA300 pathogenesis (Figure 6B) that is attributed to the high expression of Hla by this strain (Kennedy et al., 2010). In addition, a significant decrease in the weight of mice following infection with USA300 was not observed following inoculation with USA300Δ*saeR/S* or USA300Δ*saeR*-D51A (Figure 6C). These findings could not be explained by differences in inoculum concentration given to mice for each *S. aureus* strain tested (Figure 6D). As opposed to USA300Δ*saeR*-D51A, no differences could be distinguished between soft-tissue infections caused by USA300Δ*saeR*-D46A or USA300Δ*saeR*-D61A relative to USA300 (Figures 6A–C). These results demonstrate that USA300 pathogenesis during murine soft-tissue infection is dependent upon aspartic acid residue 51 of SaeR while aspartic acid residues 46 and 61 are not essential for causing disease.

## Discussion

Bacterial pathogens must recognize environmental cues and respond appropriately to cause disease. For *S. aureus*, the SaeR/S two-component system plays an important role during pathogenesis by sensing host-specific signals and up-regulating virulence gene expression in response (Geiger et al., 2008; Zurek et al., 2014). In this report, we show that the substitution of the putative SaeR phosphorylation site at aspartic acid residue 51 completely ameliorates SaeR/S-mediated virulence. Specifically, we observed a dramatic decrease in the transcript abundance of various hemolysins and leukocidins in USA300 lacking aspartic acid residue 51 of SaeR. These findings correspond to a decrease in the ability of this strain to lyse human erythrocytes and PMNs. SaeR aspartic acid residue 51 was also shown to be important for the transcription of the human MPO inhibitor SPIN and USA300 lacking SaeR aspartic acid residue 51 did not inhibit human MPO activity. Moreover, we show that the virulence of USA300Δ*saeR*-D51A during murine models of soft-tissue infection paralleled the transcript abundance of Hla in this strain, consistent with previous findings demonstrating this toxin is a major *S. aureus* virulence determinant in this model (Kennedy et al., 2010). Collectively, these findings indicate that SaeR residue 51 is essential for *S. aureus* evasion of innate immunity and support other studies that suggest only phosphorylated SaeR has DNA binding activity (Sun et al., 2010).

We also observed a more subtle decrease in the toxicity of USA300 against human erythrocytes and PMNs in the absence of the conserved aspartic acid residue 46 of SaeR. It is not clear if substitution of this residue simply perturbs the receiver domain structure of SaeR to diminish activity or if aspartic acid residue 46 plays a more direct role in the phosphorylation state of this response regulator. Regardless, a reduction in the lytic capacity of USA300Δ*saeR*-D46A suggests this conserved residue also influences SaeR function and will be further examined in future studies.

This study demonstrates that the substitution of a single amino acid residue in the SaeR response regulator of USA300 renders this highly pathogenic strain avirulent, highlighting the critical importance of two-component sensory systems for bacterial pathogenesis. Indeed, others have shown that point mutations in the dimerization interface of the histidine kinase sensor AgrC (Mairpady Shambat et al., 2016) or in the P2 promoter region of the Agr two-component system (Villaruz et al., 2009) have a profound effect on the lytic capacity and colonization potential of *S. aureus* while a point mutation in the histidine kinase sensor LiaS of *Streptococcus pyogenes* contributes to the carrier phenotype of this bacterium (Flores et al., 2017). Interestingly, no natural mutations of aspartic acid residue 51 could be identified in over 8,000 *S. aureus* genomes, suggesting this putative phosphorylation site is imperative for the fitness of this pathogen. Taken together, these studies indicate that novel therapeutic strategies that inhibit the activation of this highly conserved response regulator would impede the expression of multiple virulence factors and effectively block different aspects of pathogenesis resulting in clearance of *S. aureus* by innate immune mechanisms.

## Author Contributions

TN and JV contributed to the conception and design of this study. TN, TB, ES, FG, JD, MC, KP, LC, and BK performed the experiments and data analysis. TN and JV wrote and prepared the manuscript for submission. All authors read and approved this manuscript.

## Conflict of Interest Statement

The authors declare that the research was conducted in the absence of any commercial or financial relationships that could be construed as a potential conflict of interest.

## Footnotes

1 https://www.ebi.ac.uk/Tools/msa/clustalo/

2 https://github.com/ekg/freebayes

3 http://ftp.ncbi.nih.gov
