# Supplementary figures and images for: Aspartic Acid Residue 51 of SaeR Is Essential for Staphylococcus aureus Virulence
Source: Front Microbiol. 2018 Dec 14;9:3085. doi: 10.3389/fmicb.2018.03085 (PMC6302044; doi:10.3389/fmicb.2018.03085)

# Supplemental Figure 1

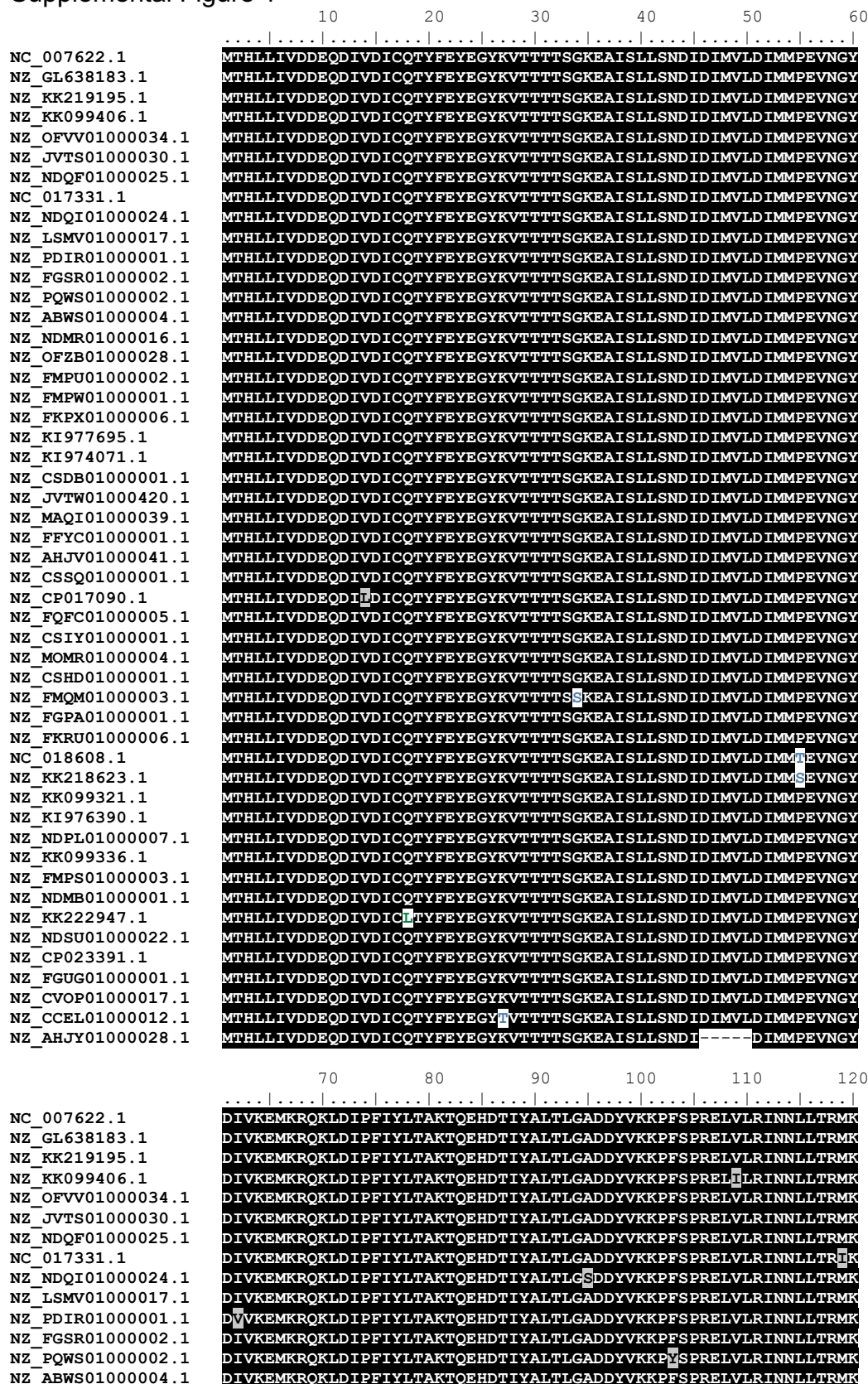

[illegible][illegible]

[illegible][illegible]

Supplement: Supplementary file 1 [file Data_Sheet_1.PDF]
