# Supplementary material for: Aspartic Acid Residue 51 of SaeR Is Essential for Staphylococcus aureus Virulence
Source: Front Microbiol. 2018 Dec 14;9:3085. doi: 10.3389/fmicb.2018.03085 (PMC6302044; doi:10.3389/fmicb.2018.03085)
Supplement: Supplementary file 2 [file Data_Sheet_2.pdf]

Supplementary Table 1

Primer	Sequence
saeR-Fwd-attB1	5'- GGG GAC AAG TTT GTA CAA AAA AGC AGG CCA TTG CTT GCG TAA TTT CCG -3'
saeR-Rvs-attB2	5'- GGG GAC CAC TTT GTA CAA GAA AGC TGG GTG CTC ACG TCA TCT TC -3'
saeR-D46A-fwd	5'- CTT TAC TAT CAA ATG ATA TTG CGA TCA TGG TAC TTG ATA TCA TG -3'
saeR-D46A-rvs	5'- CAT GAT ATC AAG TAC CAT GAT CGC AAT ATC ATT TGA TAG TAA AG -3'
saeR-D51A-fwd	5'- GAT ATT GAT ATC ATG GTA CTT GCG ATC ATG ATG CCA GAA GTT AAT G-3
saeR-D51A-rvs	5'- CAT TAA CTT CTG GCA TCA TGA TCG CAA GTA CCA TGA TAT CAA TAT C -3'
saeR-D61A-fwd	5'- GAT GCC AGA AGT TAA TGG TTA CGC GAT TGT CAA AGA AAT GAA AAG -3'
saeR-D61A-rvs	5'- CTT TTC ATT TCT TTG ACA ATC GCG TAA CCA TTA ACT TCT GGC ATC -3'
spn-Fwd-attB1	5'- GGG GAC CAC TTT GTA CAA GAA AGC TGG GTC CTT TCA GGT CCC CAT CAA TAT GC -3'
spn-Rvs-attB2	5' GGG GAC AAG TTT GTA CAA AAA AGC AGG CGG TTC AGG TAT TAT TAA AGG CC 3'
spn-check-Fwd	5'- CTA ACG GAT TCC CGA TTA CCT G -3'
spn-check-rvs	5'- CGT CCA CAT CCC TTT GTC AC -3'
saePQRS_fwd	5'- GAC AAA AAG GGG AAT CTA AGT GGT C -3'
saePQRS_rvs	5'- CAT TTT TAG CCC CTG CCA TTT TC -3'
saePQRS-Seq1	5'- GTG TAA TGT TAC AGT CAT CGT AGT TC -3'
saePQRS-Seq2	5'- CAT GGG CTA AGT TTT GAA TCA GTT C -3'
saePQRS-Seq3	5'- GTC TTG TTC ATC ATC CAC GAT C -3
saePQRS-Seq4	5'- GAA CAG GAA GTG TTT GAG CTC -3'
saePQRS-Seq5	5'- GTA GTC AAC CAT TGC GAT TTC TTT AC -3'
saeR-Xho-rvs	5'- ACC ACC CTC GAG CTC GAT ACG ACG CCA ATA ATG -3'
saeR-EcoRI-fwd	5'- ACC ACC GAA TTC GAG GTC GTA AGA ACA GAG GTG -3'
