# Supplementary material for: Aspartic Acid Residue 51 of SaeR Is Essential for Staphylococcus aureus Virulence
Source: Front Microbiol. 2018 Dec 14;9:3085. doi: 10.3389/fmicb.2018.03085 (PMC6302044; doi:10.3389/fmicb.2018.03085)
Supplement: Supplementary file 3 [file Data_Sheet_3.PDF]

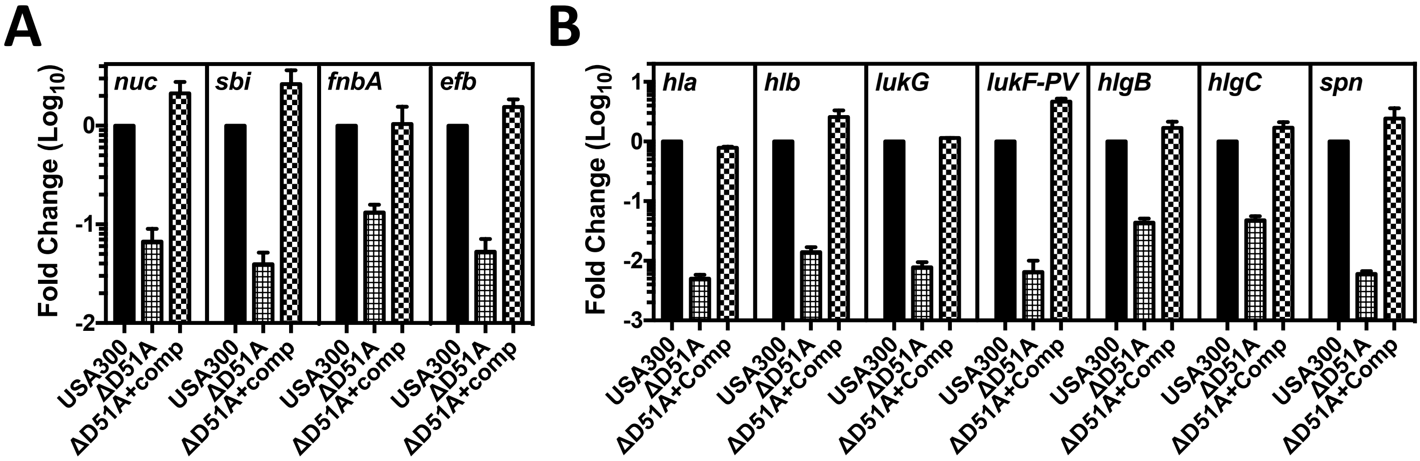

Supplementary Fig 2. Expression of wt SaeR rescues the transcription of numerous virulence genes in USA300 with an aspartic acid to alanine substitution at SaeR residue 51. Taqman® RT-PCR analysis of USA300, a USA300 genomic point mutant that confers an aspartic acid to alanine substitution at SaeR residue 51 (ΔD51A), and ΔD51A complemented with a plasmid expressing wt SaeR during growth *in vitro*. Transcriptional analysis was performed at **A)** mid-exponential growth for nuclease (*nuc)*, the second binder of IgG (*sbi*), fibronectin-binding protein A (*fnbA)*, and the extracellular fibrinogen-binding protein (*efb*) or at **B)** early-stationary growth for α-hemolysin (*hla*), β-hemolysin (*hlb*), leukocidin subunit G (*lukG)*, the Panton-Valentine leukocidin subunit F (*lukF-PV*), γ-hemolysin component B (*hlgB),* γ-hemolysin component C (*hlgC)*, and the staphylococcal peroxidase inhibitor (*spn*). All panels show the mean ± SEM of at least two separate experiments and are presented as fold change relative to USA300 wt.
